# Correction: Pushchina et al. Ultrastructural Characteristics of the Juvenile Chum Salmon (*Oncorhynchus keta*) Cerebellum: Interneuron Composition, Neuro–Glial Interactions, Homeostatic Neurogenesis, and Synaptic Plasticity. *Int. J. Mol. Sci.* 2025, *26*, 11123

**DOI:** 10.3390/ijms27083552

**Published:** 2026-04-16

**Authors:** Evgeniya V. Pushchina, Evgeniya E. Vekhova, Eugenia A. Pimenova, Anna V. Akhmadieva, Mariya E. Bykova

**Affiliations:** 1 A.V. Zhirmunsky National Scientific Center of Marine Biology, Far Eastern Branch, Russian Academy of Sciences, 690041 Vladivostok, Russia; evekhova@imb.dvo.ru (E.E.V.); epimenova@imb.dvo.ru (E.A.P.); mbykova@imb.dvo.ru (M.E.B.); 2 Research and Manufacturing Company “Home of Pharmacy”, 245, Zavodskaya St., Kuzmolovskiy t.s., Vsevolozhsk District, Leningrad Oblast, 188663 Saint Petersburg, Russia; akhmadieva.av@doclinika.ru

## Addition of Authors

“Eugenia A. Pimenova, Anna V. Akhmadieva” were not included as authors in the original publication [1]. The corrected Author Contributions and Acknowledgments statement appears here. The authors state that the scientific conclusions are unaffected. This correction was approved by the Academic Editor. The original publication has also been updated.

**Author Contributions:** Conceptualization, E.V.P.; methodology, E.E.V., E.A.P. and A.V.A.; software, E.E.V., E.A.P. and A.V.A.; validation, E.V.P., E.E.V., E.A.P. and A.V.A.; formal analysis, E.V.P.; investigation, E.V.P.; resources, E.E.V.; data curation, M.E.B.; writing—original draft preparation, E.V.P.; writing—review and editing, M.E.B.; visualization, E.E.V., E.A.P. and A.V.A.; supervision, E.V.P.; project administration, E.V.P. All authors have read and agreed to the published version of the manuscript.
**Acknowledgments:** We are grateful to the Far Eastern Center for Electron Microscopy of the Zhirmunsky National Scientific Center of Marine Biology Far Eastern Branch, Russian Academy of Sciences (NSCMB FEB RAS) for providing access to the AxioImager Z2 (Carl Zeiss, Jena, Germany), Leica UC7 ultramicrotome (Germany) and Zeiss Sigma 300 VP transmission electron microscope (Carl Zeiss, Cambridge, UK).
